# Postoperative Outcomes Among Sodium-Glucose Cotransporter 2 Inhibitor Users

**DOI:** 10.1001/jamasurg.2025.0940

**Published:** 2025-04-30

**Authors:** Roberta Teixeira Tallarico, Bocheng Jing, Kaiwei Lu, Shweta Amy Chawla, Yanting Luo, Anusha Badathala, Catherine L. Chen, Arthur W. Wallace, Matthieu Legrand

**Affiliations:** 1 Department of Anesthesia and Perioperative Care, University of California, San Francisco; 2 Department of Anesthesiology, Washington University School of Medicine in St Louis, St Louis, Missouri; 3 Division of Geriatrics, University of California, San Francisco; 4 Northern California Institute for Research and Education, San Francisco; 5 School of Medicine, University of California, San Francisco; 6 Philip R. Lee Institute for Health Policy Studies, University of California, San Francisco; 7 Veterans Affairs Medical Center, San Francisco, California

## Abstract

**Importance:**

Case reports and small retrospective studies have suggested that there is an increased risk of postoperative euglycemic ketoacidosis (eKA) and acute kidney injury (AKI) among patients using sodium-glucose cotransporter 2 inhibitors (SGLT2i) preoperatively. However, there has not been a representative assessment of the risks of these agents among patients undergoing surgery.

**Objective:**

To evaluate the risk of postoperative eKA, AKI, and mortality within 30 days after surgery among preoperative long-term SGLT2i users compared with nonusers.

**Design, Settings, and Participants:**

This is a multicenter, propensity-matched, retrospective case-control study from the Veterans Affairs Health Care System (VAHCS) National Registry performed from January 1, 2014, to December 31, 2022. Adult patients using SGLT2i preoperatively who underwent inpatient surgical procedures were compared with a 1:5 matched control group using propensity score matching, including the patient’s demographic characteristics, comorbidities, and surgical characteristics. Data analysis was performed from June 2023 to August 2024.

**Exposure:**

Long-term use of SGLT2i, defined as having more than 3 fills of outpatient prescription or less than a 180-day gap of the last fill according to the VAHCS pharmacy registries.

**Main Outcomes and Measures:**

The primary outcome was the rate of postoperative eKA among SGLT2i users vs control patients. Secondary outcomes included postoperative AKI and 30-day mortality after surgery.

**Results:**

Among 462 968 patients undergoing surgery, 7448 SGLT2i users (mean [SD] age, 67.7 [8.1] years; 7204 [96.7%] male) and 455 520 nonusers (mean [SD] age, 65.8 [11.0] years; 424 785 [93.3%] male) were identified. After propensity score matching, 7439 patients were identified as SGLT2i users and compared with 33 489 control patients. SGLT2i use was associated with an increased risk of eKA (odds ratio [OR], 1.11; 95% CI, 1.05-1.17) but reduced risks of perioperative AKI (OR, 0.69; 95% CI, 0.62-0.78) and 30-day mortality (OR, 0.70; 95% CI, 0.55-0.88). The mortality rate 30 days after surgery was 1.1% among SGLT2i users vs 1.6% among control patients. The median hospital length of stay among the patients presenting with eKA increased by 3 days (median [IQR], 6 [3-10] days for those with eKA vs 3 [2-6] days for those without eKA).

**Conclusions and Relevance:**

Patients treated with SGLT2i had a small but significantly higher risk of postoperative eKA but lower risks of postoperative AKI and 30-day mortality.

## Key Points

**Question:**

Is treatment with sodium-glucose cotransporter 2 inhibitors (SGLT2i) associated with rates of postoperative euglycemic ketoacidosis (eKA), acute kidney injury (AKI), and mortality after surgery?

**Finding:**

In this multicenter case-control study, the risk of perioperative eKA was 11% higher for patients using SGLT2i than their matched counterparts; however, there was a 31% reduction in postoperative AKI and a 30% reduction in 30-day mortality.

**Meaning:**

SGLT2i use was associated with a small but significantly higher risk of postoperative eKA but lower risks of postoperative AKI and mortality within 30 days after surgery.

## Teaser

This case-control study evaluates whether treatment with sodium-glucose cotransporter 2 inhibitors is associated with rates of postoperative euglycemic ketoacidosis, acute kidney injury, and mortality within 30 days after surgery.

## Introduction

Sodium-glucose cotransporter 2 inhibitors (SGLT2i), or gliflozins, are antidiabetic agents primarily approved by the US Food and Drug Administration to treat type 2 diabetes (T2D) since 2013.^1,2^ SGLT2i have also been shown to improve outcomes among patients with chronic kidney disease, heart failure, and coronary artery disease.^1,3,4,5,6,7,8,9,10,11,12^

However, clinical trials have reported cases of SGLT2i-associated euglycemic ketoacidosis (eKA) presenting with mildly elevated glucose levels.^13,14,15,16^ The underlying mechanism appears multiple but involves a reduced insulin secretion with SGLT2i, enhanced lipolysis, and, in turn, hepatic ketogenesis.^17^ The eKA risk may be increased during the perioperative period, given the reduction in oral carbohydrate intake during this time and the effect of surgical stress, which can increase insulin requirements and metabolic demand.^2,3,5,14,18^ This increased risk is also evidenced in the increasing number of case reports and case series of patients with postoperative eKA associated with SGLT2i use.^14,16,18,19,20,21,22,23,24,25,26,27,28,29,30,31,32^ Authors of these reports have raised concerns about the potentially severe consequences of persistent or untreated eKA. However, case reports and case series are prone to admission, selection, and reporting bias.^5,15,21,25,33^ Therefore, the actual risks of eKA and postoperative acute kidney injury (AKI) and death among SGLT2i users is unknown.

To address this gap in knowledge, we performed a multicenter, propensity-matched, retrospective case-control study to evaluate the risks of postoperative eKA and secondary clinical outcomes, including AKI and mortality, among SGLT2i users compared with matched nonusers.

## Methods

### Study Design and Database

This is a multicenter, retrospective, case-control study examining clinical outcomes after surgical inpatient procedures, using propensity-matched observational data collected from the Veterans Affairs Surgical Quality Improvement Program in the Veterans Affairs Health Care System (VAHCS). The available information includes patients’ demographic characteristics, pharmacy, and mortality registries. Data were extracted from Medical Statistical Analysis System and Corporate Data Warehouse (CDW) files in the VA [Veterans Affairs] Informatics and Computing Infrastructure. Institutional review board approval for the study was obtained from the University of California, San Francisco and Veterans Affairs Central Institutional Review Board. The study was exempt from requiring informed consent due to exclusive use of deidentified data. This study followed the Reporting of Studies Conducted Using Observational Routinely Collected Data (RECORD) extension of the Strengthening the Reporting of Observational Studies in Epidemiology (STROBE) reporting guidelines. Data analysis was performed from June 2023 to August 2024.

### Study Population

Using the Medical Statistical Analysis System files, we identified patients aged 18 years and older who underwent surgical procedures requiring a hospital stay of more than 24 hours between 2014 and 2022. We excluded cases classified as American Society of Anesthesiologists Physical Status class VI ambulatory surgery and subsequent cases where the same patient underwent repeated operations within 90 days of the initial procedure; in such cases, only the first procedure was considered.^34^ However, multiple procedures for the same patient were included if there was a 90-day interval between them. Inpatient cases without laboratory tests within the first 7 days after surgery were also excluded, as were cases with serum lactate levels higher than 27 mg/dL after surgery (to convert to millimoles per liter, multiply by 0.111).

To identify adult surgery patients who were consistently prescribed SGLT2i preoperatively, we looked for patients with any prescription for empagliflozin, canagliflozin, and dapagliflozin, administered either as monotherapy or in combination with other widely prescribed antidiabetic medications, such as biguanides and gliptins (Table 1). We defined long-term preoperative SGLT2i use as 3 or more fills of outpatient prescriptions within 3 months prior to surgery or less than a 180-day gap since the most recent fill. The information on the time of the last dose of SGLT2i was not available on this dataset. We categorized the SGLT2i users and control cohort by the surgical specialty performing the index procedure (ie, neurosurgery, orthopedic surgery, urology, cardiac surgery, thoracic surgery, general surgery, vascular surgery, plastic surgery, ophthalmology, gynecologic surgery, otolaryngology, podiatry, proctology). We also identified comorbidities and cardiovascular and nephrological risk factors from inpatient and outpatient diagnosis files in the CDW using *International Statistical Classification of Diseases and Related Health Problems, Tenth Revision*^35^ diagnosis codes (eTable 1 in Supplement 1). To define the control group, we applied a propensity score–matching (PSM) technique, including relevant variables such as patients’ demographic characteristics, comorbidities, and surgical characteristics.

**Table 1.** List of the US Food and Drug Administration–Approved Sodium-Glucose Cotransporter 2 Inhibitor Medications

Generic name	Commercial name
Canagliflozin	Invokana
Canagliflozin + metformin	Invokanamet
Canagliflozin + metformin XR	Invokanamet XR
Dapagliflozin	Farxiga
Dapagliflozin + metformin	Xigduo XR
Dapagliflozin + saxagliptin	Qtern
Empagliflozin	Jardiance
Empagliflozin + linagliptin	Glyxambi
Empagliflozin + metformin	Synjardy
Empagliflozin + linagliptin + metformin XR	Trijardy XR
Ertugliflozin	Steglatro
Ertugliflozin + metformin	Segluromet
Ertugliflozin + sitagliptin	Steglujan

Abbreviation: XR, extended release.

### Outcome Definitions

The primary outcome was postprocedure eKA. To determine postoperative eKA diagnosis, we used a modified definition based on serum laboratory test results from postoperative days 1 to 7, including the presence of metabolic acidosis (bicarbonate <18 mEQ/L or base excess <−5 mEQ/L [to convert to millimoles per liter, multiply by 1]), high anion gap (anion gap >12 mEq/L [to convert to millimoles per liter, multiply by 1]), and glucose levels less than 200 mg/dL (to convert to millimoles per liter, multiply by 0.555), in the absence of high lactate (lactate <27 mg/dL). While different glucose levels have been reported to define eKA, we used the most conservative one to avoid misclassification of potential decompensated diabetes as eDKA.^36^

### Secondary Outcomes

The secondary outcomes assessed in the study included AKI and in-hospital mortality within the first 30 days after the procedure. AKI was defined following the Kidney Disease: Improving Global Outcomes definition using plasma creatinine concentration criteria.^37^ Baseline serum creatinine concentration was the most recent value within 60 days before the surgical procedure. Mortality information was ascertained from VAHCS vital status files.

### Covariate Data

Patients’ demographic characteristics, anthropometric data, and medical history were reported, as were details on the documented surgical procedure (surgical specialty, anesthesia technique, surgery duration) and specific postoperative information (AKI, in-hospital 30-day mortality). Race and ethnicity were self-reported and extracted from the VA CDW. Race was categorized as American Indian or Alaska Native, Asian, Black, Hawaiian or Other Pacific Islander, White, multiple or unknown, and declined to answer; ethnicity was categorized as Hispanic or non-Hispanic. The hospital length of stay after surgery was also collected.

### Prespecified Subgroup Analysis

The type of surgery and the urgency of the case may influence the metabolic response after surgery. Therefore, we prespecified a subgroup analysis for patients undergoing cardiac surgery, as it is often associated with intense stress, a pronounced metabolic response, and prolonged procedure duration (eTable 2 in Supplement 1).

To assess the impact of the timing of SGLT2i discontinuation before surgery, we analyzed a subgroup of patients undergoing emergency procedures, considering them as control patients due to the inability to withhold the medication preoperatively (eTable 3 in Supplement 1).

### Statistical Analysis

The exploratory data analysis included demographic, clinical, and surgical information from the overall cohort as well as for the SGLT2i users and control group before and after PSM. Categorical variables were summarized using counts and percentages, while continuous variables were reported as medians and IQRs. Standardized mean differences (SMDs) were reported before and after PSM.

The primary goal was to investigate the perioperative incidence of eKA in the cohort of SGLT2i users compared with matched control patients, using PSM to balance the treatment and control groups based on the baseline covariates. Patients using SGLT2i were compared with a 1:5 matched control group. The optimal matching algorithm was used, with an SMD less than 0.1 considered indicative of well-matched variables. The odds ratios (ORs) of eKA, AKI, and mortality in 30 days were independently calculated and presented with a 95% CI. No power analysis was performed prior to the data extraction as the sample size was driven by the number of patients treated with SGLT2i, the uncertain incidence of eKA, and the anticipated large sample size of the population enrolled expected to provide high power. Additional information can be found in the eMethods in Supplement 1. The analyses were conducted using SAS version 9.4 software (SAS Institute Inc); the figures were generated using R version 4.4 software (R Foundation).

## Results

### Patient Characteristics

**Figure 1.**
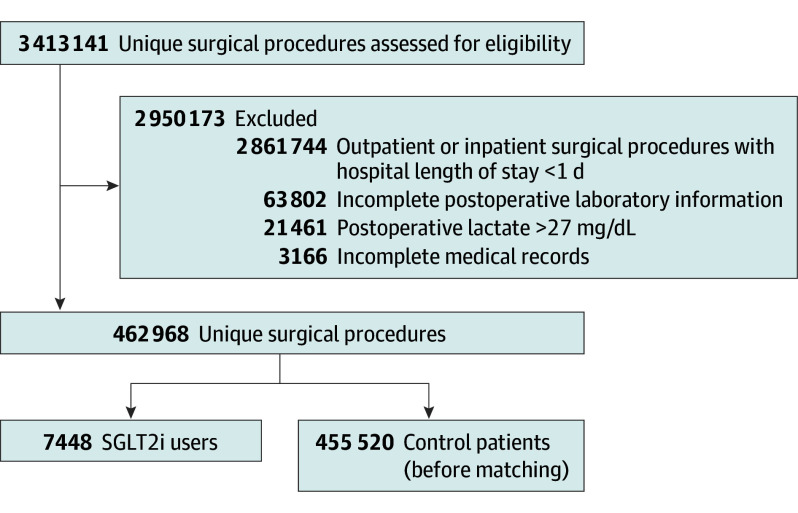
Flow Diagram Showing the Number of Individuals Included in the Study Population To convert lactate to millimoles per liter, multiply by 0.111. SGLT2i indicates sodium-glucose cotransporter 2 inhibitor.

Among 462 968 surgical patients who met the inclusion criteria for this study, we identified 7448 SGLT2i users (mean [SD] age, 67.7 [8.1] years; 7204 [96.7%] male) and 455 520 control patients (mean [SD] age, 65.8 [11.0] years; 424 785 [93.3%] male) (Figure 1 and Table 2). After PSM, our final cohort included 40 928 patients (7439 SGLT2i users and 33 489 control patients). Patients were predominantly male (7196 [96.7%] in the SGLT2i group vs 32 288 [96.4%] in the control group; SMD, 0.018), White (5476 [73.6%] in the SGLT2i group vs 24 402 [72.9%] in the control group; SMD, 0.049), and older than 65 years (mean [SD] age, 67.7 [8.1] years in the SGLT2i group vs 67.9 [8.8] years in the control group; SMD, −0.022). The patients’ characteristic distributions before and after PSM can be found in eFigure 1 in Supplement 1. The median body mass index (calculated as weight in kilograms divided by height in meters squared) was 30.6 (IQR, 26.7-34.8) for SGLT2i users and 30.3 (IQR, 26.4-34.7) for control patients. Most patients were American Society of Anesthesiologists Physical Status class III (5039 [67.7%] in the SGLT2i group vs 22 909 [68.4%] in the control group; SMD, 0.000). The most common comorbidities included diabetes (7004 [94.2%] in the SGLT2i group vs 31 316 [93.5%] in the control group; SMD, 0.027), hypertension (6966 [93.6%] in the SGLT2i group vs 31 228 [93.2%] in the control group; SMD, 0.016), peripheral vascular disease (2457 [33.0%] in the SGLT2i group vs 10 435 [31.2%] in the control group; SMD, 0.040), and congestive heart failure (2193 [29.5%] in the SGLT2i group vs 8698 [26.0%] in the control group; SMD, 0.078) (Table 2).

**Table 2.** Summary and Clinical Characteristics of the Study Population

Characteristic	Overall (n = 462 968)			1:5 Matching (n = 40 928)		
	SGLT2i users (n = 7448)	Control patients (n = 455 520)	SMD	SGLT2i users (n = 7439)	Control patients (n = 33 489)	SMD
Age, mean (SD), y	67.7 (8.1)	65.8 (11.0)	0.199	67.7 (8.1)	67.9 (8.8)	−0.022
Sex, No. (%)						
Female	244 (3.3)	30 735 (6.7)	0.160	243 (3.3)	1201 (3.6)	0.018
Male	7204 (96.7)	424 785 (93.3)		7196 (96.7)	32 288 (96.4)	
Race, No. (%)^a^						
American Indian or Alaska Native	60 (0.8)	4587 (1.0)	0.150	60 (0.8)	312 (0.9)	0.049
Asian	63 (0.8)	2122 (0.5)		63 (0.8)	257 (0.8)	
Black	1346 (18.1)	87 639 (19.2)		1345 (18.1)	6394 (19.1)	
Hawaiian or Other Pacific Islander	75 (1.0)	3732 (0.8)		75 (1.0)	330 (1.0)	
White	5483 (73.6)	336 932 (74.0)		5476 (73.6)	24 402 (72.9)	
Multiple or unknown	421 (5.7)	205 08 (4.5)		420 (5.6)	1794 (5.4)	
Ethnicity, No. (%)^a^						
Hispanic	528 (7.1)	25 845 (5.7)	0.058	527 (7.1)	2355 (7.0)	0.003
Non-Hispanic	6920 (92.9)	429 675 (94.3)		6912 (92.9)	31 134 (93.0)	
BMI, median (IQR)	30.6 (26.7-34.8)	28.6 (24.9-32.8)	0.307	30.6 (26.7-34.8)	30.3 (26.4-34.7)	0.021
ASA Physical Status classification, No. (%)						
I-II	104 (1.4)	56 209 (12.3)	0.488	103 (1.4)	468 (1.4)	0.000
III	5046 (67.7)	305 880 (67.1)		5039 (67.7)	22 909 (68.4)	
IV-V	2298 (30.9)	93 431 (20.5)		2297 (30.9)	10 112 (30.2)	
Comorbidities, No. (%)						
Diabetes	7012 (94.1)	133 040 (29.2)	1.795	7004 (94.2)	31 316 (93.5)	0.027
Hypertension	6974 (93.6)	309 708 (68)	0.689	6966 (93.6)	31 228 (93.2)	0.016
Congestive heart failure	2196 (29.5)	40 583 (8.9)	0.541	2193 (29.5)	8698 (26.0)	0.078
Chronic kidney disease	27 (0.4)	2929 (0.6)	0.04	27 (0.4)	164 (0.5)	−0.020
COPD	1502 (20.2)	80 979 (17.8)	0.061	1500 (20.2)	6613 (19.7)	0.010
Peripheral vascular disease	2461 (33)	71 657 (15.7)	0.412	2457 (33.0)	10 435 (31.2)	0.040
Liver disease	564 (7.6)	23 010 (5.1)	0.104	564 (7.6)	2612 (7.8)	−0.008
Home medication, No. (%)						
Metformin	4832 (64.9)	79 632 (17.5)	1.100	4829 (64.9)	20 692 (61.8)	0.065
Sulfonylurea	1918 (25.8)	38 107 (8.4)	0.475	1918 (25.8)	8346 (24.9)	0.020
Insulin	6328 (85.0)	146 989 (32.3)	1.266	6321 (85.0)	28 067 (83.8)	0.032
ACE inhibitors	4092 (54.9)	313 921 (68.9)	0.291	3353 (45.1)	15 206 (45.4)	−0.007
Angiotensin receptor blockers	2315 (31.1)	50 319 (11.0)	0.507	2312 (31.1)	8968 (26.8)	0.095
Surgical specialty, No. (%)						
General	1364 (18.3)	109 738 (24.1)	0.468	1363 (18.3)	6398 (19.1)	0.059
Cardiac	716 (9.6)	28 147 (6.2)		716 (9.6)	3076 (9.2)	
Gynecology	11 (0.1)	3718 (0.8)		11 (0.1)	49 (0.1)	
Neurosurgery	428 (5.7)	28 441 (6.2)		427 (5.7)	1959 (5.8)	
Ophthalmology	3 (<0.1)	29 (<0.1)		2 (<0.1)	7 (<0.1)	
Orthopedic	1492 (20.0)	123 567 (27.1)		1491 (20.0)	6855 (20.5)	
Otolaryngology	135 (1.8)	9753 (2.1)		135 (1.8)	580 (1.7)	
Plastic	53 (0.7)	3561 (0.8)		53 (0.7)	276 (0.8)	
Proctology	2 (<0.1)	143 (<0.1)		2 (<0.1)	9 (<0.1)	
Thoracic	250 (3.4)	20 484 (4.5)		250 (3.4)	1171 (3.5)	
Urology	475 (6.4)	45 473 (10.0)		475 (6.4)	2373 (7.1)	
Oral	2 (<0.1)	420 (0.1)		2 (<0.1)	9 (<0.1)	
Podiatry	386 (5.2)	6345 (1.4)		386 (5.2)	1540 (4.6)	
Peripheral vascular	2131 (28.6)	75 701 (16.6)		2126 (28.6)	9187 (27.4)	
Procedure class						
Emergency	625 (8.4)	35 651 (7.8)	0.021	625 (8.4)	2855 (8.5)	−0.004
Nonemergency	6823 (91.6)	419 869 (92.2)		6814 (91.6)	30 634 (91.5)	
Anesthesia type, No. (%)						
General	6237 (83.7)	399 676 (87.7)	0.233	6232 (83.8)	28 180 (84.1)	0.055
Epidural	9 (0.1)	842 (0.2)		9 (0.1)	32 (0.1)	
Local	19 (0.3)	1048 (0.2)		19 (0.3)	85 (0.3)	
Monitored	663 (8.9)	16 987 (3.7)		660 (8.9)	2788 (8.3)	
Regional	140 (1.9)	5097 (1.1)		140 (1.9)	620 (1.9)	
Spinal	380 (5.1)	31 825 (7.0)		379 (5.1)	1784 (5.3)	
Other	0	45 (<0.1)		0	0	
Case duration, min						
Median (IQR)	138 (88-228)	139 (93-215)	0.024	138 (88-228)	136 (86-222)	0.024
>120, No. (%)	4324 (58.1)	274 860 (60.3)	0.047	4321 (58.1)	19 281 (57.6)	0.010

Abbreviations: ACE, angiotensin-converting enzyme; ASA, American Society of Anesthesiologists; BMI, body mass index (calculated as weight in kilograms divided by height in meters squared); COPD, chronic obstructive pulmonary disease; SGLT2i, sodium-glucose cotransporter 2 inhibitors; SMD, standardized mean difference.

^a^ Race and ethnicity data were self-reported and extracted from the Veterans Affairs Corporate Data Warehouse.

The most common procedures included peripheral vascular operations (2126 [28.6%] in the SGLT2i group vs 9187 [27.4%] in the control group), orthopedic operations (1491 [20.0%] in the SGLT2i group vs 6855 [20.5%] in the control group), and general surgery procedures (1363 [18.3%] in the SGLT2i group vs 6398 [19.1%] in the control group). Cardiac operations were performed in 716 SGLT2i users (9.6%) and 3076 control patients (9.2%) (eTable 2 in Supplement 1). Most patients in the SGLT2i group (6232 [83.8%]) and control group (28 180 [84.1%]) underwent general anesthesia during the procedures. Emergency procedures were performed in 625 SGLT2i users (8.4%) and 2855 control patients (8.5%) (eTable 3 in Supplement 1). The median surgical case duration was 138 minutes (IQR, 88-228 minutes) among the SGLT2i group and 136 minutes (IQR, 86-222 minutes) among the control group.

In the SGLT2i group, empagliflozin was the most common SGLT2i (7413 patients [99.7%]). There was a progressive increase in the number of SGLT2i users undergoing surgery throughout the years in this cohort, and 80% of the patients in our cohort underwent surgery between 2020 and 2022 (eFigure 2 in Supplement 1). The time of holding SGLT2i prior to procedures was not available in this cohort.

### eKA Incidence

**Figure 2.**
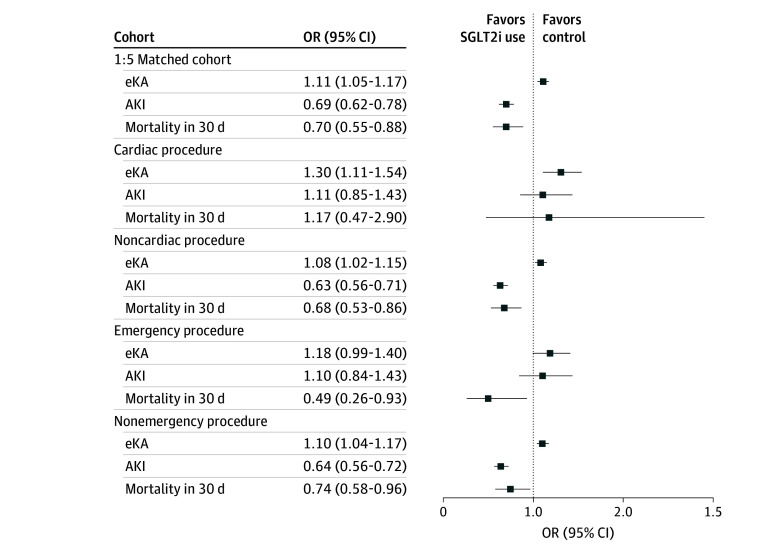
Postoperative Risk of Euglycemic Ketoacidosis (eKA), Acute Kidney Injury (AKI), and 30-Day Mortality Among Sodium-Glucose Cotransporter 2 Inhibitor (SGLT2i) Users vs Control Patients Representation of clinical outcomes for the SGTL2i users and control patients, including eKA, AKI, and 30-day mortality. Additionally, the difference between the overall cohort outcomes and the subgroup analysis for cardiac and emergency procedures is shown. The study included 7439 SGLT2i users (716 underwent cardiac surgery and 6723, noncardiac surgery; 625 underwent emergency procedures and 6814, nonemergency procedures) and 33 489 control patients (3076 underwent cardiac surgery and 30 413, noncardiac surgery; 3480 underwent emergency procedures and 30 009, nonemergency procedures). Additional information on subgroups can be found in eTable 2 and eTable 3 in Supplement 1. OR indicates odds ratio.

Overall, eKA occurred in 2210 SGLT2i users (29.7%) and 9255 control patients (27.6%) (OR, 1.11; 95% CI, 1.05-1.17) (Figure 2). Patients who developed eKA had longer hospital lengths of stay compared with those who did not (median, 6 [IQR, 3-10] days vs 3 [IQR, 2-6] days, respectively).

Among the 3792 patients who underwent cardiac surgery, the incidence of eKA was 56.4% among SGLT2i users vs 49.8% among control patients (OR, 1.30; 95% CI, 1.11-1.54). For patients undergoing noncardiac surgery, the eKA incidence was 26.8% among the SGLT2i users vs 25.3% among controls (OR = 1.08; 95% CI, 1.02-1.15) (Figure 2; eTable 2 in Supplement 1). Results were unchanged after excluding podiatry cases (n = 1926; eTable 4 in Supplement 1).

Among patients undergoing emergency surgery, eKA occurred in 46.2% of SGLT2i users vs 42.1% of control patients (emergent cases: OR, 1.18; 95% CI, 0.99-1.40; nonemergent cases: OR, 1.10; 95% CI, 1.04-1.17) (Figure 2; eTable 3 in Supplement 1).

### Postoperative AKI Incidence

Postoperative AKI occurred in 380 patients (5.1%) in the SGLT2i group vs 2410 control patients (7.1%) (OR, 0.69; 95% CI, 0.62-0.78) (Figure 2). After cardiac surgery, AKI occurred in 11.3% of the SGLT2i group vs 10.3% of control patients (after cardiac surgery: OR, 1.11; 95% CI, 0.85-1.43; noncardiac surgery: OR, 0.63; 95% CI, 0.56-0.71) (Figure 2; eTable 2 in Supplement 1). After emergency surgery, AKI occurred in 12% of the SGLT2i group vs 11.3% of controls (after emergency surgery: OR, 1.10; 95% CI, 0.84-1.43; nonemergency surgery: OR, 0.64; 95% CI, 0.56-0.72) (Figure 2; eTable 3 in Supplement 1).

### Postoperative Mortality

The 30-day mortality rate after surgery was 1.1% among SGLT2i users (n = 81) vs 1.6% among control patients (n = 521) (OR, 0.70; 95% CI, 0.55-0.88) (Figure 2). The OR for mortality was 1.17 (95% CI, 0.47-2.90) in patients who underwent cardiac surgery vs 0.68 (95% CI, 0.53-0.86) in patients with noncardiac operations (Figure 2; eTable 2 in Supplement 1). The OR for mortality was 0.49 (95% CI, 0.26-0.93) in patients who underwent emergency surgery vs 0.74 (95% CI, 0.58-0.96) in patients who received nonemergent operations (Figure 2; eTable 3 in Supplement 1).

## Discussion

In this large, national, retrospective, multicenter, case-control study of preoperative SGLT2i use and incidence of postoperative eKA in surgical patients, we identified an 11% increased risk of eKA among SGLT2i users across all surgical procedures, a 30% increased risk of eKA following cardiac surgical procedures, and an 18% increased risk of eKA following emergency surgical procedures compared with matched control patients. We also observed a lower risk of postoperative AKI and mortality among SGLT2i users compared with control patients.

Gliflozins have several physiological mechanisms that can lead to eKA, including increasing glucose and insulin requirements while lowering the insulin to glucagon ratio, which can lead to a metabolic shift toward lipid oxidation, enhancing lipolysis and consequently increasing the hepatic production of ketoacids. SGLT2i use can thus promote the development of metabolic acidosis. A systematic review of previously published clinical trials, which included 16 clinical trials and 31 256 patients (18 956 SGLT2i users and 12 300 in the placebo group), showed a relative risk of 3.70 (95% CI, 2.58-5.29) for eKA in SGLT2i users compared with individuals in the placebo group.^13^ The risk for eKA has also been associated with periods of metabolic stress, such as periods of fasting, illness, and dehydration, which are common for patients in perioperative settings.^15,18^ In a systematic review of case reports of 77 patients, surgery was reported as the most common event associated with eKA (20% of SGLT2i users).^13^

The incidence of eKA in SGLT2i users in perioperative settings has been described in previously published small case series and retrospective studies.^13,15,16,24^ A retrospective matched cohort study, which included 155 patients, described the incidence of postoperative metabolic acidosis with euglycemia as 22.6% among SGLT2i users vs 8.1% in the matched control cohort.^15^ In our cohort, we identified an eKA incidence of 29.6% among surgical patients using SGLT2i, which is higher than the incidences previously reported. The difference may stem from the varying populations enrolled, differences in the eKA definitions used, and potential discrepancies in the timing of SGLT2i discontinuation before surgery.

The consequences of developing perioperative eKA are not fully understood; however, perioperative eKA may necessitate escalated interventions (ie, insulin administration or continuous dextrose infusion), which could be associated with an increased risk of prolonged hospitalization or unplanned admission to an intensive care unit.^24,32,38^ As expected, we observed an increase in hospital length of stay among the patients with eKA diagnosis in this study.

The eKA incidence among patients undergoing cardiac surgical procedures is higher than the incidence in another single-center retrospective study, which included 1654 cardiac surgical procedures and reported an eKA incidence of 15%.^39^ Our cohort includes patients across different VAHCS centers, which comprise a more diverse population that may be subject to differences in clinical management.^39^ This includes potential short durations of holding the medication prior to surgery compared with other cohorts. Emergency surgical procedures are usually not preceded by suspending SGLT2i prior to surgery. We observed a nonsignificant increase in the eKA risk among SGLT2i after emergent surgery. The imprecision of the effect size might be related to the smaller sample size. While these results are consistent with the findings of previous studies, the relatively small difference in the effect size for eKA incidence in SGLT2i users compared with control patients suggests that the impact of holding SGLT2i before surgery on the occurrence of eKA is very uncertain.

Another concern in SGLT2i use was a potential increased risk of AKI. The increased urinary sodium and glucose excretion due to SGLT2i use results in osmotic diuresis, which can precipitate hypovolemia.^11,13,15,40^ Hypovolemia is among the causes of prerenal kidney disease in the perioperative setting.^11,41^ However, in this study, we observed a lower risk of AKI in patients treated with SGLT2i compared with control patients. The potential reasons are uncertain but may involve an extension of the renal protective effects of SGLT2i during the postoperative period. The mechanisms of renal protection of SGLT2i are thought to involve better glycemic control, decreased sodium reabsorption, and reduced inflammation, oxidative stress, and fibrosis.^6,11^ Additionally, SGLT2i reduces albuminuria and cardiovascular risk, further protecting kidney function. This renoprotective effect may provide increased kidney reserve in the event of an injury (ie, surgical stress), which may consequently protect patients from developing AKI. Renoprotective effects of SGLT2i in the perioperative setting could have far-reaching implications given the annual volume of patients undergoing surgical procedures and the burden of postoperative AKI.^42,43^

As the development of AKI has been consistently associated with a higher risk of mortality in acutely ill patients, which could be due to remote organ injury after AKI, we also explored the association between SGLT2i use and the risk of postoperative death.^44,45^ While the overall risk of death in this cohort was low, we did observe a lower risk of death among SGLT2i users compared with control patients, consistent with previous studies.^5^

### Limitations

This study has several limitations. First, we could not establish direct causality in our findings given the observational nature of the study, although our matching approach aimed to limit potential confounders. Next, we could not determine the clinical impact of holding SGLT2i medication use prior to surgery, and the exact timing of the last administration for patients was unknown. The US Food and Drug Administration recommends suspension of SGLT2i for 72 to 108 hours before elective surgery to prevent the risk of postoperative eKA.^1,5,40^ While most institutions follow this recommendation, the subgroup analysis of emergency surgical procedures (where the medication is likely not suspended) offers insights into the association between clinical outcomes and SGLT2i use when the medication is likely not suspended. Another consideration is that the absence of ketone measurements limited our eKA diagnostic criteria. However, in a perioperative scenario, ketone measurements are not routinely included in decision-making and clinical management. The true sensitivity of ketonuria as a biomarker to define eKA remains controversial within the scientific community.^40^ Additionally, the population assisted by the VAHCS predominantly comprises individuals who identify as White, male, and older than 60 years, which limits the generalizability of the results.

## Conclusions

In this retrospective matched case-control study, patients treated with SGLT2i had a slightly but statistically significantly higher risk of postoperative eKA compared with control patients. It also showed a lower risk of AKI and 30-day mortality.

## Notes

Supplement 1. **eMethods.** Supplemental Methods **eTable 1.** List of International Classification of Diseases (ICD-10) Diagnosis Codes **eTable 2.** Summary and Clinical Characteristics of the Subgroup of Cardiac Surgeries **eTable 3.** Summary and Clinical Characteristics of the Subgroup of Emergency Surgeries **eTable 4.** Sensitivity Analysis of the Risk of Euglycemic Ketoacidosis (eKA), Acute Kidney Injury (AKI), and Mortality after Exclusion of Podiatry Cases **eFigure 1.** Distribution of Patients’ Characteristics Before and After Propensity Score Matching **eFigure 2.** Distribution of Surgical Cases on the VAHCS Throughout the Years, for the SGLT2i Users and Controls

Supplement 2. Data Sharing Statement
