# Supplementary material for: Postoperative Outcomes Among Sodium-Glucose Cotransporter 2 Inhibitor Users
Source: JAMA Surg. 2025 Apr 30;160(6):681–9. doi: 10.1001/jamasurg.2025.0940 (PMC12044541; doi:10.1001/jamasurg.2025.0940)
Supplement: Supplement 1. — eMethods. Supplemental Methods eTable 1. List of International Classification of Diseases (ICD-10) Diagnosis Codes eTable 2. Summary and Clinical Characteristics of the Subgroup of Cardiac Surgeries eTable 3. Summary and Clinical Characteristics of the Subgroup of Emergency Surgeries eTable 4. Sensitivity Analysis of the Risk of Euglycemic Ketoacidosis (eKA), Acute Kidney Injury (AKI), and Mortality after Exclusion of Podiatry Cases eFigure 1. Distribution of Patients’ Characteristics Before and After Propensity Score Matching eFigure 2. Distribution of Surgical Cases on the VAHCS Throughout the Years, for the SGLT2i Users and Controls [file jamasurg-e250940-s001.pdf]

Supplemental Online Content

Tallarico RT, Jing B, Lu K, et al. Postoperative outcomes among sodium-glucose cotransporter 2 inhibitors users. *JAMA Surg*. Published online April 30, 2025. doi:10.1001/jamasurg.2025.0940

eMethods. Supplemental Methods
eTable 1. List of *International Classification of Diseases* (*ICD-10*) Diagnosis Codes
eTable 2. Summary and Clinical Characteristics of the Subgroup of Cardiac Surgeries
eTable 3. Summary and Clinical Characteristics of the Subgroup of Emergency Surgeries
eTable 4. Sensitivity Analysis of the Risk of Euglycemic Ketoacidosis (eKA), Acute Kidney Injury (AKI), and Mortality after Exclusion of Podiatry Cases
eFigure 1. Distribution of Patients' Characteristics Before and After Propensity Score Matching
eFigure 2. Distribution of Surgical Cases on the VAHCS Throughout the Years, for the SGLT2i Users and Controls

This supplemental material has been provided by the authors to give readers additional information about their work.

© 2025 Tallarico RT et al. *JAMA Surgery*.

eMethods. Supplemental Methods

Propensity score calculation and matching algorithm

For the propensity score calculation and matching algorithm, we used a greedy matching algorithm with replacement to perform 1:5 propensity score matching (PSM). The propensity score was estimated using a logistic regression model, adjusting for confounders listed in the Love plot (eFigure 1 in the Supplement). Each SGLT2i user (case) was matched with up to five controls based on their propensity scores, ensuring comparable baseline characteristics. We applied a caliper of 0.25 on the logit scale of the propensity score (default in SAS) as matching standard. Additionally, to further control for confounding, we exactly matched on diabetes and hypertension status, ensuring that cases and controls shared the same baseline conditions for these key variables. After matching, we assessed the quality of balance using standardized mean differences (SMDs), which were displayed in the Love plot. All covariates had SMDs < 0.1, indicating excellent balance between the matched groups. After matching, we did not include additional adjustments for matched covariates in the outcome model, as the matching process ensured balance. The final logistic regression model included SGLT2i exposure as the only independent variable.

Odds Ratio computation

For the odds ratio (OR) calculation, we applied a logistic regression model to the matched cohort. The primary predictor was SGLT2i use**,** and since the propensity score matching ensured covariate balance, no further adjustments for additional covariates were made in the outcome model. Odds ratios were directly obtained from this logistic regression.

© 2025 Tallarico RT et al. *JAMA Surgery*.

eTable 1. List of *International Classification of Diseases* (*ICD-10*) Diagnosis Codes

**Comorbidities**	***ICD-9* codes**	***ICD-10* codes**
Diabetes	250.0x, 250.1x, 250.2x, 250.3x, 250.4x, 250.5x, 250.7x, 250.8x, 250.9x, 250.x0, 250.x1	E10.2 - E10.8, E11.2 - E11.8, E12.2 - E12.8, E13.2 - E13.8, E14.2 - E14.8<br>E10.0, E10.1, E10.9, E11.0, E11.1, E11.9, E12.0, E12.1, E12.9, E13.0, E13.1, E13.9, E14.0, E14.1, E14.9
Hypertension	401.0,401.10, 401.9	I10.x
Congestive heart failure	428.x	I09.9, I11.0, I13.0, I13.2, I25.5, I42.0, I42.5 - I42.9, I43.x, I50.x, P29.0
Chronic kidney disease	585.9	N18.9
Chronic obstructive pulmonary disease	490-496	I27.8, I27.9, J40.x - J47.x, J60.x - J67.x, J68.4, J70.1, J70.3
Peripheral vascular disease	440-449	I70.x, I71.x, I73.1, I73.8, I73.9, I77.1, I79.0, I79.2, K55.1, K55.8, K55.9, Z95.8, Z95.9
Liver disease	571.2, 571.4-571.6, 572	B18.x, I85.x, I86.4, I98.2, K70.x, K71.1, K71.3 - K71.5, K71.7, K72.x - K74.x, K76.0, K76.2 - K76.9, Z94.4

Comorbidities defined by *ICD-9* and *ICD-10* codes listed in this study to calculate the propensity score matching.

© 2025 Tallarico RT et al. *JAMA Surgery*.

eTable 2. Summary and Clinical Characteristics of the Subgroup of Cardiac Surgeries

Characteristic	Overall			1:5 Matching		
**No. of patients**	SGTT2i user (N=716)	Controls (N=28 147)	SMD[a]	SGTT2i user (N=716)	Controls (N=3076)	SMD[a]
**Age, mean (SD), y**	68.2 (8.1)	68.2 (8.8)	-0.01	68.2 (8.1)	68.7 (8.2)	-0.063
**Sex male, n (%)**	701 (97.9)	27646 (98.2)	0.507	701 (97.9)	3035 (98.7)	-0.0587
**Race, n (%)**			0.137			0.137
White	549 (76.7)	22 948 (81.5)		549 (76.7)	2480 (80.6)	
Black	96 (13.4)	3131 (11.1)		96 (13.4)	329 (10.7)	
American Indian/Alaskan Native	9 (1.3)	235 (0.8)		9 (1.3)	28 (0.9)	
Asian	13 (1.8)	208 (0.7)		13 (1.8)	39 (1.3)	
Hawaiian/Pacific Islander	12 (1.7)	253 (0.9)		12 (1.7)	37 (1.2)	
Multiple or unknown	37 (5.2)	1372 (4.9)		37 (5.2)	163 (5.3)	
Hispanic	57 (8)	1690 (6)	0.0768	57 (8)	232 (7.5)	0.0157
**BMI, median (SD, IQR)**	30.9 (27.5-34.9)	29.1 (25.8-33.1)	0.2921	30.9 (27.5-34.9)	30.3 (7.0, 26.4-34.7)	0.021
**ASA, n (%)**			0.1478			0.0157
1-2	0 (0)	23 (0.1)		0 (0)	0 (0)	
3	45 (6.3)	2724 (9.7)		45 (6.3)	199 (6.5)	
4-5	671 (93.7)	25400 (90.2)		671 (93.7)	2877 (93.5)	
**Comorbidities, n (%)**						
Diabetes	666 (93)	11 107 (39.5)	1.374	666 (93)	2664 (86.6)	0.2131
Hypertension	697 (97.3)	25548 (90.8)	0.281	697 (97.3)	2967 (96.5)	0.0514
Congestive heart failure	324 (45.3)	6202 (22)	0.5069	324 (45.3)	1427 (46.4)	-0.0229
Chronic kidney disease	2 (0.3)	221 (0.8)	-0.0696	2 (0.3)	10 (0.3)	-0.0083
COPD	170 (23.7)	7115 (25.3)	-0.0357	170 (23.7)	763 (24.8)	-0.0248
Peripheral vascular disease	124 (17.3)	3467 (12.3)	0.1411	124 (17.3)	497 (16.2)	0.0311
Liver disease	48 (6.7)	1146 (4.1)	0.1168	48 (6.7)	195 (6.3)	0.0148
**Home Medication, n (%)**						
Metformin	464 (64.8)	6887 (24.5)	0.8878	464 (64.8)	1816 (59)	0.119
Sulfonylurea	230 (32.1)	3593 (12.8)	0.477	230 (32.1)	824 (26.8)	0.1172
Insulin	675 (94.3)	24626 (87.5)	0.2373	675 (94.3)	2924 (95.1)	-0.0349
ACE inhibitors	368 (51.4%)	12812 (45.5)	0.1178	368 (51.4)	1538 (50)	0.0279

© 2025 Tallarico RT et al. *JAMA Surgery*.

Angiotensin receptor blockers	260 (36.3)	4635 (16.5)	0.4621	260 (36.3)	1004 (32.6)	0.0773
**Emergency Surgery (%)**	159 (22.2)	3834 (13.6)	0.2253	159 (22.2)	655 (21.3)	0.0221
**Case duration, median (IQR)**	283.5 (213.0-345.5)	272 (212.0-335.0)	0.0301	283.5 (213.0-345.5)	276 (204.5-345.0)	0.0543
**Case Duration > 120 minutes (%)**	609 (85.1)	25579 (90.9%)	-0.1796	609 (85.1)	2625 (85.3)	-0.0079

a.SMD: standardized mean deviation

ACE inhibitors: angiotensin-converting enzyme inhibitors. ASA: American Society of Anesthesiologists Physical Status. BMI: body mass index, COPD: chronic obstructive pulmonary disease. SGLT2i: sodium-glucose cotransporter 2 inhibitors.

© 2025 Tallarico RT et al. *JAMA Surgery*.

eTable 3. Summary and Clinical Characteristics of the Subgroup of Emergency Surgeries

Characteristic	Overall			1:5 Matching		
No. of patients	SGTT2i user (N=625)	Controls (N=35651)	SMD[a]	SGTT2i user (N=625)	Controls (N=3480)	SMD[a]
**Age, mean (SD), y**	67.3 (8.7)	65.4 (13.5)	0.167	67.3 (8.7)	67.7 (9.4)	-0.046
**Sex male, n (%)**	615 (98.4)	33688 (94.5)	0.212	615 (98.4)	2782 (97.4)	0.067
**Race, n (%)**			0.124			
White	485 (77.6)	26488 (74.3)		485 (77.6)	2067 (72.4)	0.15
Black	91 (14.6)	6524 (18.3)		91 (14.6)	522 (18.3)	
American Indian/Alaskan Native	7 (1.1)	426 (1.2)		7 (1.1)	28 (1)	
Asian	10 (1.6)	249 (0.7)		10 (1.6)	32 (1.1)	
Hawaiian/Pacific Islander	8 (1.3)	322 (0.9)		8 (1.3)	38 (1.3)	
Multiple or unknown	421 (5.7)	20508 (4.5)		421 (5.7)	168 (5.9)	
Hispanic, n (%)	43 (6.9)	2543 (7.1)	-0.009	43 (6.9)	233 (8.2)	-0.049
**BMI, median (SD, IQR)**	30.8 (15.1-55.2)	27.5 (23.7-31.9)	0.437	30.8 (15.1-55.2)	29.5 (25.8-33.9)	
**ASA, n (%)**			0.520			0.041
1-2	104 (1.4)	56209 (12.3)		104 (1.4)	26 (0.9)	
3	5046 (67.7)	305880 (67.1)		5046 (67.7)	1248 (43.7)	
4-5	2298 (30.9)	93431 (20.5)		2298 (30.9)	1581 (55.4)	
**Comorbidities, n (%)**						
Diabetes	589 (94.2)	10562 (29.6)	1.782	589 (94.2)	2624 (91.9)	0.092
Hypertension	588 (94.1)	23691 (66.5)	0.740	588 (94.1)	2633 (92.2)	0.074
Congestive heart failure	250 (40)	4661 (13.1)	0.640	250 (40)	983 (34.4)	0.115
Chronic kidney disease	8 (1.3)	747 (2.1)	-0.063	8 (1.3)	19 (0.7)	0.063
COPD	129 (20.6)	6975 (19.6)	0.026	129 (20.6)	613 (21.5)	-0.02
Peripheral vascular disease	218 (34.9%)	6060 (17%)	0.416	218 (34.9%)	265 (9.3)	0.106
Liver disease	65 (10.4)	2336 (6.6)	0.138	65 (10.4)	265 (9.3)	0.038
**Home Medication, n (%)**						
Metformin	377 (60.3)	5336 (15)	1.059	377 (60.3)	1622 (56.8)	0.071
Sulfonylurea	178 (28.5)	2764 (7.8)	0.558	178 (28.5)	681 (23.9)	0.105
Insulin	553 (88.5)	13828 (38.8)	1.206	553 (88.5)	2540 (89)	-0.015
ACE inhibitors	284 (45.4)	10908 (30.6)	0.309	284 (45.4)	1302 (45.6)	-0.003
Angiotensin receptor blockers	211 (33.8)	3672 (10.3)	0.590	211 (33.8)	742 (26)	0.17

© 2025 Tallarico RT et al. *JAMA Surgery*.

**Surgical Specialty, n (%)**			0.617			0.318
General Surgery	202 (32.3)	17 773 (49.9)		202 (32.3)	871 (30.5)	
Cardiac Surgery	159 (25.4)	3834 (10.8)		159 (25.4)	655 (22.9)	
Gynecology	0 (0)	65 (0.2)		0 (0)	0 (0)	
Neurosurgery	16 (2.6)	1360 (3.8)		16 (2.6)	99 (3.5)	
Ophthalmology	1 (0.2)	3 (0)		1 (0.2)	1 (0)	
Orthopedic Surgery	45 (7.2)	4434 (12.4)		45 (7.2)	323 (11.3)	
Otolaryngology	3 (0.5)	475 (1.3)		3 (0.5)	31 (1.1)	
Plastic Surgery	2 (0.3)	205 (0.6)		2 (0.3)	20 (0.7)	
Proctology	0 (0)	15 (0)		0 (0)	1 (0)	
Thoracic Surgery	2 (0.3)	436 (1.2)		2 (0.3)	21 (0.7)	
Urology	24 (3.8)	1193 (3.3)		24 (3.8)	77 (2.7)	
Oral Surgery	1 (0.2)	68 (0.2)		1 (0.2)	3 (0.1)	
Podiatry	31 (5)	728 (2)		31 (5)	197 (6.9)	
Peripheral Vascular Surgery	139 (22.2)	5062 (14.2)		139 (22.2)	556 (19.5)	
**Anesthesia Type**			0.2283			0.184
General	556 (89)	33313 (93.4)		556 (89)	2573 (90.1)	
Epidural	3 (0.5)	16 (0)		3 (0.5)	1 (0)	
Local	4 (0.6)	79 (0.2)		4 (0.6)	7 (0.2)	
Monitored	43 (6.9)	1367 (3.8)		43 (6.9)	211 (7.4)	
Regional	12 (1.9)	219 (0.6)		12 (1.9)	38 (1.3)	
Spinal	7 (1.1)	653 (1.8)		7 (1.1)	25 (0.9)	
Other	0 (0)	4 (0)		0 (0)	0 (0)	
**Case duration, median (IQR)**	104.0 (58.0-237.0)	92.0 (57.0-154.0)	0.2806	104.0 (58.0-237.0)	103.0 (58.0-217.0)	0.073
**Case Duration > 120 minutes (%)**	273 (43.7)	12799 (35.9)	0.1594	273 (43.7)	1239 (43.4)	0.006

a.SMD: standardized mean deviation

ACE inhibitors: angiotensin-converting enzyme inhibitors. ASA: American Society of Anesthesiologists Physical Status. BMI: body mass index, COPD: chronic obstructive pulmonary disease. SGLT2i: sodium-glucose cotransporter 2 inhibitors.

© 2025 Tallarico RT et al. *JAMA Surgery*.

eTable 4. Sensitivity Analysis of the Risk of Euglycemic Ketoacidosis (eKA), Acute Kidney Injury (AKI), and Mortality after Exclusion of Podiatry Cases

Outcome	1:5 matching without Podiatry[a] N=39 002	Cardiac subgroup[b] N=3 792	Non-cardiac subgroup N=35 210	Emergency N=3 252	Non-Emergency N=35 750
**eKA**	1.13 (1.07, 1.20)	1.30 (1.11, 1.54)	1.10 (1.04, 1.17)	1.18 (0.99, 1.42)	1.13 (1.06, 1.20)
**AKI**	0.70 (0.63, 0.79)	1.11 (0.85, 1.43)	0.64 (0.56, 0.72)	1.11 (0.85, 1.46)	0.64 (0.57, 0.73)
**Mortality in 30 days**	0.74 (0.58, 0.94)	1.17 (0.47, 2.90)	0.72 (0.56, 0.92)	0.28, 0.98)	0.79 (0.61, 1.02)

a. Sensitivity analysis excluding Podiatry cases
b. Cardiac subgroup not expected to change after Podiatry exclusion

© 2025 Tallarico RT et al. *JAMA Surgery*.

eFigure 1. Distribution of Patients' Characteristics Before and After Propensity Score Matching

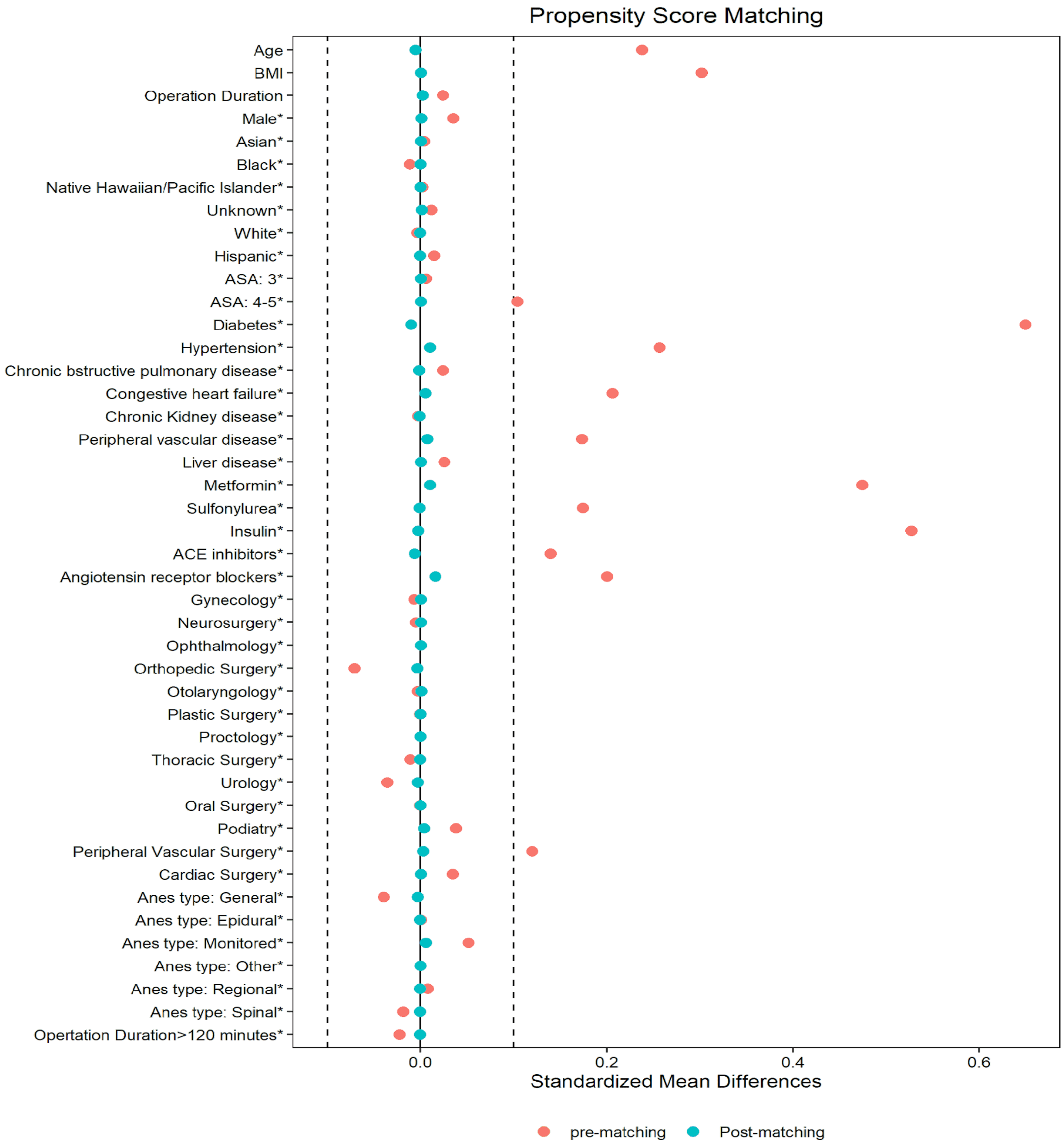

Propensity score matching graphic to demonstrate the distribution of SGLT2i users and controls before and after matching.

© 2025 Tallarico RT et al. *JAMA Surgery*.

eFigure 2. Distribution of Surgical Cases on the VAHCS Throughout the Years, for the SGLT2i Users and Controls

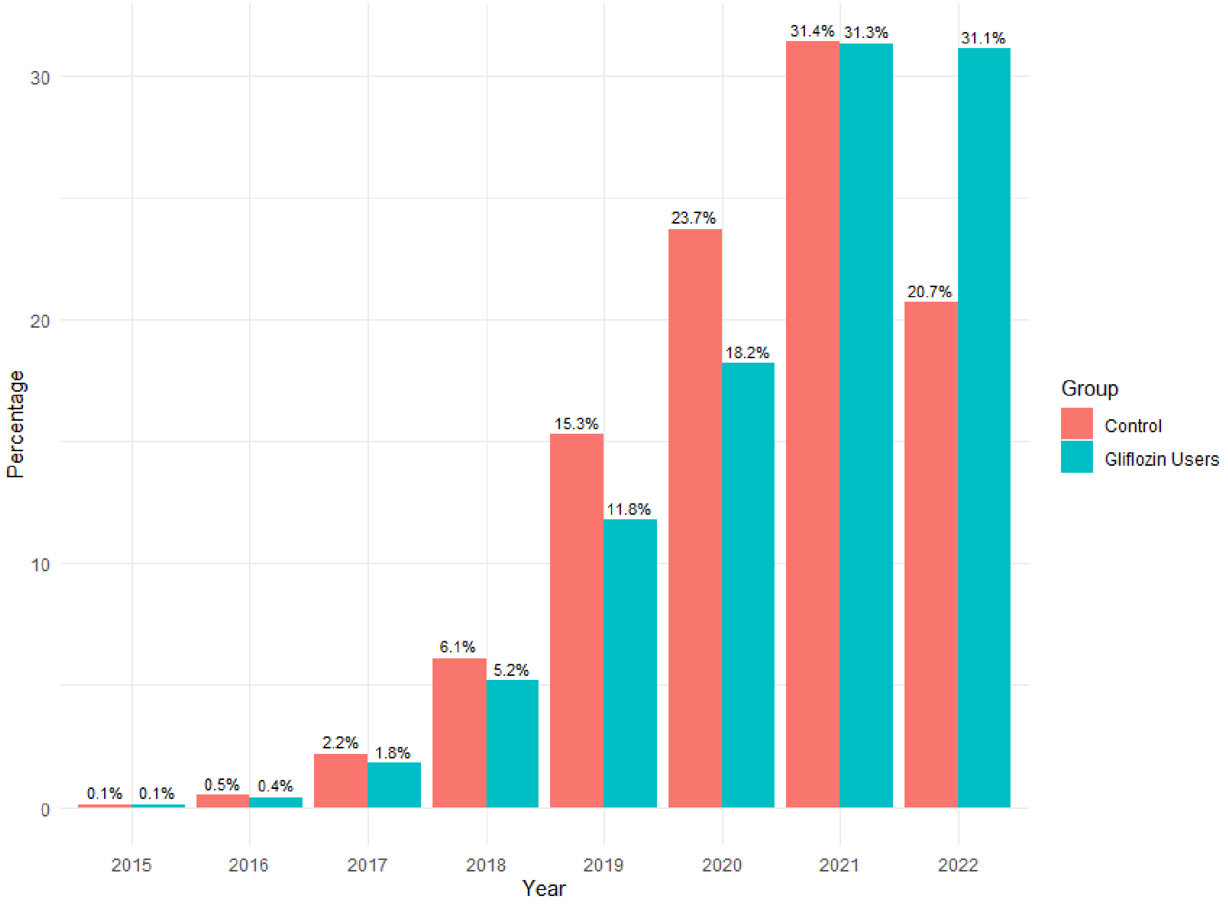

Number of SGLT2i users and controls by year, reporting the progressive increase in SGLT2i use across the years.

© 2025 Tallarico RT et al. *JAMA Surgery*.
