# Supplementary material for: Postoperative Outcomes Among Sodium-Glucose Cotransporter 2 Inhibitor Users
Source: JAMA Surg. 2025 Apr 30;160(6):681–9. doi: 10.1001/jamasurg.2025.0940 (PMC12044541; doi:10.1001/jamasurg.2025.0940)
Supplement: Supplement 2. — Data Sharing Statement [file jamasurg-e250940-s002.pdf]

Data Sharing Statement

Tallarico. Postoperative Outcomes Among Sodium-Glucose Cotransporter 2 Inhibitor Users. *JAMA Surg*. Published April 30, 2025. doi:10.1001/jamasurg.2025.0940

Data
Data available: No

Additional Information
Explanation for why data not available: The VA database is protected and can only be accessed by authorized personnel.
